# Dietary Inclusion of Hydrolyzed *Debaryomyces hansenii* Yeasts Modulates Physiological Responses in Plasma and Immune Organs of Atlantic Salmon (*Salmo salar*) Parr Exposed to Acute Hypoxia Stress

**DOI:** 10.3389/fphys.2022.836810

**Published:** 2022-03-28

**Authors:** Byron Morales-Lange, Brankica Djordjevic, Ashwath Gaudhaman, Charles McLean Press, Jake Olson, Liv Torunn Mydland, Luis Mercado, Mónica Imarai, Mathieu Castex, Margareth Øverland

**Affiliations:** ^1^Department of Animal and Aquaculture Sciences, Faculty of Biosciences, Norwegian University of Life Sciences, Ås, Norway; ^2^Department of Preclinical Sciences and Pathology, Faculty of Veterinary Medicine, Norwegian University of Life Sciences, Ås, Norway; ^3^Department of Animal and Dairy Sciences, University of Wisconsin, Madison, WI, United States; ^4^Grupo de Marcadores Inmunológicos, Facultad de Ciencias, Instituto de Biología, Pontificia Universidad Católica de Valparaíso, Valparaíso, Chile; ^5^Departamento de Biología, Facultad de Química y Biología, Centro de Biotecnología Acuícola, Universidad de Santiago de Chile, Santiago, Chile; ^6^Lallemand SAS, Blagnac, France

**Keywords:** blood, distal intestine, gill, immunological markers, RNAseq, short-term stress, yeast-based products

## Abstract

Stress related to salmon aquaculture practices (handling, sub-optimal nutrition, diseases, and environmental problems) may compromise fish welfare. This study describes the effects of two hydrolyzed *Debaryomyces hansenii* yeast-based products (LAN4 and LAN6) on physiological and immune responses of Atlantic salmon (*Salmo salar*) parr exposed to short hypoxia stress. A commercial-like diet (control diet: CD) and two experimental diets (CD supplemented with 0.1% of either component LAN4 or LAN6) were fed to fish for 8 weeks. At the end of the feeding experiment, fish were exposed to 1-min hypoxia and samples were collected at 0, 1, 3, 6, 12, and 24 h post-stress. Results showed that plasma cortisol reached a peak at 1 h post-stress in CD and LAN6 groups, whereas no significant increase in cortisol levels was detected in the LAN4 group. Moreover, the LAN6 group enhanced IL-10 responses to hypoxia, when compared to the control and LAN4 group. This suggests a regulation of immunosuppressive profiles in fish fed LAN4. Hypoxia stress increased TNFα in all groups, which indicates that fish may compensate for the short-term stress response, by modulating innate immune molecules. The apparent suppression of hypoxia responses in the LAN4 group coincided with the detection of differences in goblet cells size and Muc-like proteins production in DI; and upregulation (1 h post-stress) of pathways related to oxygen transport, hemoglobin complex, and glutathione transferase activity and the downregulation of fatty acid metabolism (6 h post-stress) in gills. To conclude, a 1-min hypoxia stress exposure affects the response to stress and immunity; and *D. hansenii*-based yeast products are promising components in functional aquafeeds for salmon due to their ability to counteract possible consequences of hypoxic stress.

## Introduction

Aquaculture is a continuously expanding global industry. However, the rapid growth and the subsequent intensification in this sector causes increased stress incidences and increased risk of infectious diseases, which leads to large economic losses (Kiron, 2012; Food and Agriculture Organization of the United Nations, 2020).

Different processes related to salmonid farming such as transportation, grading, vaccination, and handling, where fish are taken out of the water or are exposed to low dissolved oxygen concentrations, can cause short-term stress, which deteriorates fish welfare (Braithwaite and Ebbesson, 2014; Peterson et al., 2019) due to negative synergistic effects on the physiological response of the fish (e.g., immune response) (Catalán et al., 2019; Reid et al., 2019).

Preventive measures and good husbandry practices are important for the sustainable growth of aquaculture. Nutrition and the use of functional feeds can help to alleviate stress. They can prevent immune suppression, as certain feed components can reduce the risk of diseases, and improve health and overall fish welfare. Modulation of the immune system by functional ingredients has previously been shown in several teleost fish such as Chinook salmon (*Onchoryhnchus tshawytscha*), rainbow trout (*Oncorhynchus mykiss*), African catfish (*Clarius gariepinus*), gilthead sea bream (*Sparus aurata*), European sea bass, (*Dicentrarchus labrax*), tilapia (*Oreochromis niloticus*), turbot (*Scophthalmus maximus*), and Indian carp (*Labeo rohita*). Moreover, in Atlantic salmon (*Salmo salar*), it has already been reported that functional ingredients or additives can beneficially impact health by modulating the respiratory burst of head kidney macrophage, controlling profiles associated with soybean meal-induced enteritis (SBMIE), and improving the robustness of the fish against various infectious agents (e.g., *Neoparamoeba perurans* and piscine myocarditis virus) (Torrecillas et al., 2012; Meena et al., 2013; Martinez-Rubio et al., 2014; Fuchs et al., 2017; Mullins et al., 2020; Agboola et al., 2021a; Rawling et al., 2021).

Among different functional ingredients, microbial ingredients (e.g., non-*Saccharomyces* yeast such as *Cyberlindnera jadinii*, *Blastobotrys adeninivorans*, and *Wickerhamomyces anomalus* or its cell components) are gaining increased interest in aquafeeds during recent years (Torrecillas et al., 2012; Ochangco et al., 2016; Øverland and Skrede, 2017; Glencross et al., 2020; Rawling et al., 2021). Yeast contains a wide range of microbe-associated molecular patterns (MAMPs) (e.g., α-glucan, β-glucan, α-mannan, and nucleic acids) (Navarrete and Tovar-Ramírez, 2014), which act on host pattern recognition receptors (PRRs) such as Toll-like receptors (TLRs) and C-type lectin receptors (CLRs), activating cells of the immune system (Whyte, 2007). Furthermore, these molecules can modulate the immune response of salmonids, which induces both local effects in the intestine (strengthening the intestinal barrier and increasing resistance to infectious pathogens) and systemic effects, by regulating pro- and anti-inflammatory cytokines, effector molecules, and coordinating antigen-presenting cells (Schmitt et al., 2015; Sahlmann et al., 2019; Morales-Lange et al., 2021).

A promising non-*Saccharomyces* yeast candidate as a functional ingredient for aquafeeds is *Debaryomyces hansenii.* This yeast has been isolated from different sources such as soil, seawater, foods, and clinical samples (Wrent et al., 2015). In different animal groups (from invertebrates to higher vertebrates), it has been described that *D. hansenii* possesses probiotic properties, which induces gut microbiota modulation [e.g., increasing operational taxonomic units (OTUs) of intestinal bacteria and promoting high colonization of the digestive tract], enhanced animal survival against pathogen challenges such as *Amyloodinium ocellatum* and *Vibrio anguillarum*, and stimulating the humoral immune response of the host (Reyes-Becerril et al., 2008; Caruffo et al., 2015; Angulo et al., 2018, 2020). Furthermore, selected inactivated fractions from this yeast by heat or hydrolysis (e.g., cell wall components and polyamines) have also shown immunomodulatory properties, increasing both phagocytic ability, and the production of nitric oxide in leukocytes, in addition to inducing the expression of immune-related genes such as NFκB, IL-1β, and TNFα (Angulo et al., 2018, 2020).

The main objective of this study was to evaluate the effect of two different hydrolyzed *D. hansenii* yeast-based products on plasma biomarkers and immune organs of Atlantic salmon after exposure to short-term hypoxia stress. This knowledge will facilitate the inclusion of bioactive components based on *D. hansenii* in functional feeds for salmonids, with the potential to modulate stress-related responses in fish exposed to challenging conditions.

## Materials and Methods

### Experimental Design

The experiment was carried out at the Center for Sustainable Aquaculture at the Norwegian University of Life Sciences, Ås, Norway. All animals were treated according to the laws and regulations for experiments on live animals in the EU (Directive 2010/637EU) and Norway (FOR-2015-06-18-761).

A total of 540 Atlantic salmon pre-smolts (35 g initial body weight; GEN-innOva^®^ GAIN from AquaGen AS; Trondheim, Norway) were randomly distributed in nine circular tanks (300-L fiberglass tanks with averaged 14.5°C recirculated fresh water at a flow rate keeping the oxygen level above 80% saturation). The experiment lasted 7 weeks, starting with 5 weeks of winter regime 8/16 h (L/D) and ending with 2 weeks of 24-h light. All units were controlled by OxyGuard water quality monitoring and control systems for aquaculture (OxyGuard International A/S, Denmark). No significant changes in water quality were detected.

Each tank was randomly allocated one of three experimental diets (in triplicate): a commercial-like diet (control diet: CD), and two experimental diets, where the control diet was supplemented with 0.1% of either compound LAN4 or LAN6 (hydrolyzed *D. hansenii*-based products from Lallemand SAS, France). The diets were produced by extrusion and subsequent vacuum coating with fish oil at the Centre for Feed Technology, Norwegian University of Life Sciences (Ås, Norway). The composition of each diet is described in Table 1, and the morphological characterization, composition, and origin of both hydrolyzed *D. hansenii*-based products are shown in Table 2.

**TABLE 1:** Diets composition.

Ingredient (%)	CD	LAN4	LAN6
Fish meal^a^	39	39	39
Soy protein concentrate^b^	24.67	24.67	24.67
Wheat gluten^c^	3.34	3.34	3.34
Wheat^d^	11.8	11.8	11.8
Fish oil^e^	8.09	8.09	8.09
Rapeseed oil^f^	9	9	9
Premix^g^	2.85	2.85	2.85
*Debaryomyces hansenii* (LAN4)^h^	0	0.1	0
*Debaryomyces hansenii* (LAN6)^h^	0	0	0.1
Water correction	1.24	1.14	1.14
Yttrium oxide^i^	0.01	0.01	0.01

*^a^LT Fish meal, Norsildmel AS, Bergen, Norway.*

*^b^Soy protein concéntrate, Tradkon SPC HC-200, Sojaprotein, Becej, Serbia.*

*^c^Wheat gluten, Amilina AB, Panevezys, Lithuania.*

*^d^Wheat, Norgesmøllene, Bergen, Norway.*

*^e^Fish oil (28 % EPA + DHA), Nordsildmel AS, Bergen, Norway.*

*^f^Rapeseed oil, AAK, Karlshamn, Sweden.*

*^g^Premix (vitamineral-p-AA-kolin), BioMar AS, Norway.*

*^h^Debaryomyces hansenii, Lallemand, France.*

*^i^Yttrium oxide, Metal Rare Earth Limited, Shenzen, China.*

**TABLE 2:** Characterization of hydrolyzed *D. hansenii*-based products.

	LAN4	LAN6
Cell size (μm)	3.2 ± 0.5	3.5 ± 0.6
Cell wall thickness (nm)	143.1 ± 35.7	119.2 ± 24.8
α-glucans (% w/w)	19.2	6.6
β–glucans (% w/w)	40.8	19.3
Mannans (% w/w)	7.4	14.8
Mannans length (nm)	83	105
Ratio βglucans/Mannans	5.5	1.3
Origin	Marine	Dairy

*% w/w, expressed as % of dry matter.*

Fish were fed 10% excess for 7 weeks using automatic belt feeders (6 h a day) with a feeding level of 2% of body weight. After 7 weeks of feeding period, 42 Atlantic salmon per dietary group were distributed into seven tanks (6 fish/tank). Fish were acclimatized and continued to feed on their respective diets for 1 more week until the exposure to an acute hypoxia stress challenge.

The acute hypoxia stress challenge applied was described by Djordjevic et al. (2021a). Briefly, for each dietary treatment, a pre-stress group was sampled, and then, short-term hypoxia was inflicted by netting the fish out of the experimental tanks for 1 min and afterward placing them back into their respective tanks. After the stressful condition, six fish per diet and sampling time (0 h: immediately after stress, 1, 3, 6, 12, and 24 h post-stress exposure) were randomly netted out, sedated using 15 mg L^–1^ MS222, previously neutralized with sodium hydrogen carbonate (EMSURE^®^ ACS, Reag.Ph Eur) and sampled for blood within 5 min from the moment they were placed into the sedation bath. Approximately 1 ml of blood was withdrawn from the caudal vein using 2-ml heparinized syringes. The blood samples were separated by centrifugation (3,000 × g; 5 min), and then, the plasma was aliquoted into 1.5-ml sterile Eppendorf tubes and stored at −80°C until analysis. Thereafter, each fish was killed by a quick blow to the head, measured for body weight, and dissected to obtain distal intestine (DI) and gill samples (from the left-hand-side second gill arch). For histology, DI samples were placed in 10% neutral buffered formalin for 48 h at room temperature and then processed according to the routine histological procedures. For molecular analysis, samples of DI and gill were immediately placed in RNA later, stored overnight at 4°C, and then kept at −80°C.

### Plasma Parameters

Plasma levels of cortisol and glucose were assayed in duplicate per sample by a competitive enzyme-linked immunosorbent assay (ELISA) Cortisol assay kit (Ab108665, Abcam) and a Glucose assay kit (Ab65333, Abcam) using a SpectraMax microplate reader (Molecular Devices) and according to the manufacturer’s instructions.

To detect immunological markers in plasma (TNFα; IL-10), an indirect ELISA was performed according to the study of Morales-Lange et al. (2021). Briefly, total proteins were quantified using the BCA Protein Assay Kit (Pierce) following the manufacturer’s instructions. Then, each plasma sample was diluted in NaHCO_3_ (60 mM pH 9.6) and seeded (by duplicate) in a 96-well plate (Nunc) at 50 ng μl^–1^ (100 μl) for overnight incubation at 4°C. After, 200 μl of Pierce Clear Milk Blocking Buffer 1x (BioRad) was incubated for 2 h at 37°C. Next, 100 μl of the primary antibody (Table 3) was added and incubated for 90 min at 37°C. After successive washes with PBST (PBS with 0.2% Tween 20), the secondary antibody was incubated for 60 min at 37°C (goat anti-mouse IgG-HRP diluted 1: 5,000). Finally, after several washes with PBST, chromagen substrate 3,3′,5,5′-tetramethylbenzidine single solution (TMB, Thermo Fisher Scientific) was added (100 μl) and incubated for 20 min (in dark) at room temperature (RT). The reaction was stopped with 50 μl of 1 N sulfuric acid and read at 450 nm on a SpectraMax microplate reader (Molecular Devices).

**TABLE 3:** Primary antibodies for indirect enzyme-linked immunosorbent assay (ELISA).

Molecule	Source	Dilution	References
TNFα	Mouse	1:500	Morales-Lange et al., 2021
IL-10	Mouse	1:500	Morales-Lange et al., 2021
Muc-like proteins	Mouse	1:400	Djordjevic et al., 2021b

### Distal Intestine

Histological sections of DI were prepared following routine histology procedures. In brief, formalin-fixed DI samples were dehydrated, embedded in paraffin wax, sectioned at 5 μm thickness, dried in an oven overnight at 37°C, and stained with hematoxylin and eosin (HE) and periodic acid Schiff (PAS). Sections were examined by light microscope (Zeiss Axio Lab.A1, Carl Zeiss, Germany) for the measurement of morphological parameters: villi simple fold length, goblet cells size, and goblet cell area according to the study of Djordjevic et al. (2021a). Furthermore, Muc-like proteins were detected in DI samples. For this, each DI sample was mechanically homogenized (with metal beads) in RIPA buffer (1:4) and then, total proteins were quantified through the BCA Protein Assay Kit. Thereafter, the indirect ELISA protocol was as follows: the samples were diluted in NaHCO_3_ (60 mM pH 9.6) and seeded by duplicate in a 96-well plate (100 μl at 50 ng μl^–1^) for overnight incubation (4°C). The next day, 200 μl of blocking buffer (1x) was incubated (2 h at 37°C), and then, the primary antibody against Muc-like proteins (Table 3) was added (100 μl) and incubated (90 min at 37°C). After successive washes with PBST, the secondary antibody (goat anti-mouse IgG-HRP diluted 1: 5,000) was incubated (60 min at 37°C) and washed (with PBST). TMB was added (100 μl) and incubated for 20 min (in dark) at RT. The reaction was stopped with 50 μl of 1 N sulfuric acid and read at 450 nm on a SpectraMax microplate reader.

### RNA Sequencing

Gill samples from CD and LAN4 groups (3 fish per diet and sampling time: pre-stress, 1 h post-stress, and 6 h post-stress) were used to determine the gene expression by RNA sequencing (RNA-seq). Briefly, total RNA was extracted using the RNeasy Mini Kit (Qiagen) following the supplier’s instructions. Each RNA sample was quantified using a NanoDrop™ 8000 spectrophotometer (NanoDrop Technologies). RNA integrity was measured by Agilent Bioanalyzer 2100 (all samples showed an RNA integrity number ≥ 7). Thereafter, RNA-seq was performed by the Norwegian Sequencing Center (UiO, Norway) using Illumina NovaSeq 6000 System (150 bp paired-end RNA sequencing).

### Data Analysis

Data analysis (means, standard deviation, and multiple comparisons test) and graphical presentation of the results were done using GraphPad Prism 8.0.2 (San Diego, CA, United States). One-way ANOVA with a Dunnett’s multiple comparison *post-hoc* test was used to compare each post-stress time point with the pre-stress group (per diet). Furthermore, Tukey’s multiple comparison test was used to compare the different diets within each time point. Differences were considered significant at *p* < 0.05.

Correlation coefficients were calculated using the corrplot package in R (Wei, 2021) among the different parameters by diet. The correlations were considered significant at *p* < 0.01 (degrees of freedom = 6).

RNA-seq raw data analysis was performed using nf-core rnaseq v3.3 (Ewels et al., 2020). Cleaned reads were aligned to *Salmo salar* genome SSAL_v3.1 (GenBank assembly accession: GCA_905237065.2). Fragment mapping was counted using featureCounts (subread v1.5.1), and differentially expressed genes (DEGs) were estimated using the SARTools R package (v1.7.3). Significant DEGs were determined when the adjusted *p*-value (padj) was < 0.05. ShinyGO v0.741 (Ge et al., 2020) was used to perform the enrichment analysis and functional classification of DEGs. Term categories (FDR < 0.05) were displayed and sorted by fold enrichment (minGSSize = 2).

## Results

### Health Status and Growth Performance

During the trial, no mortality was recorded, and no significant differences in final body weight were observed between the dietary groups (CD: 103.6 g ± 15.8, LAN4: 108.7 g ± 17.7, LAN6: 104.4 g ± 19.2).

### Plasma Biomarkers

**FIGURE 1:**
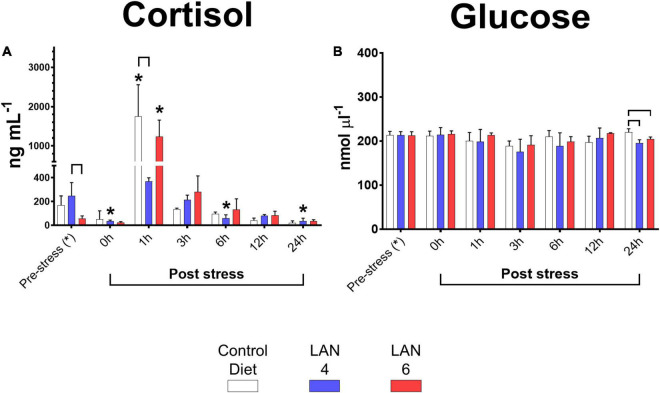
Circulating cortisol and glucose in the plasma of Atlantic salmon exposed to acute hypoxia stress. **(A)** Cortisol (ng ml^–1^). **(B)** Glucose level (nmol μl^–1^). Sampling times: pre-stress, 0 h (immediately after stress), 1, 3, 6, 12, and 24 h post-stress. In white, CD. In blue, LAN4. In red, LAN6. Each bar: *n* = 4. *: Significant differences (*p* < 0.05) compared with pre-stress group (from each diet). Bracket: significant differences (*p* < 0.05) among dietary groups within sampling time (CD, LAN4, and LAN6, respectively).

Plasma cortisol results (Figure 1A) showed that the baseline levels (pre-stress groups) were as follows: 169.08 ± 77.38 ng ml^–1^ (CD), 152.38 ± 97.67 ng ml^–1^ (LAN4), and 57.04 ± 21.58 ng ml^–1^ (LAN6). Compared to their respective baseline levels, CD and LAN6 groups showed a significant increase in cortisol at 1 h post-stress (1,749.68 ± 806.99 ng ml^–1^ and 1,238.51 ± 419.41 ng ml^–1^, respectively). In comparison, the LAN4 group did not show a significant increase in cortisol during the post-stress period; however, a significant decrease was measured in this dietary group at 0 (34.83 ± 9.54 ng ml^–1^), 6 (60.36 ± 27.50 ng ml^–1^), and 24 h (35.25 ± 24.97 ng ml^–1^) post-stress (compared to the pre-stress group). In addition, by comparing the different diet groups within the sampling time, a significantly higher level of cortisol was detected in LAN4 in the pre-stress group (compared to LAN6). Nevertheless, LAN4 had a lower cortisol level (369.78 ± 29.04 ng ml^–1^) compared to CD at 1 h post-stress.

Regarding plasma glucose levels (Figure 1B), at 24 h post-stress, both diets with the inclusion of *D. hansenii* showed lower glucose levels (LAN4: 195.59 ± 7.31 nmol μl^–1^ and LAN6: 204.19 ± 5.00 nmol μl^–1^) than CD (219.71 ± 8.24 nmol μl^–1^).

**FIGURE 2:**
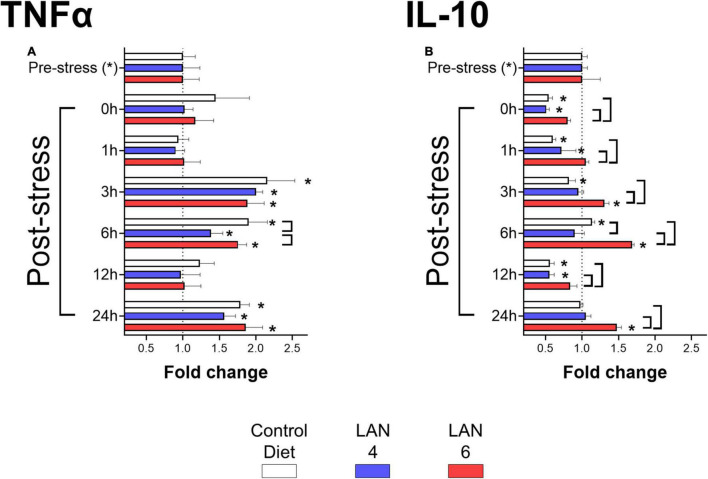
Immunological biomarkers in plasma of Atlantic salmon exposed to acute hypoxia stress. Sampling times: pre-stress, 0 h (immediately after stress), 1, 3, 6, 12, and 24 h post-stress. In white: CD. In blue: LAN4. In red: LAN6. Each bar: *n* = 4. *: Significant differences (*p* < 0.05) compared with pre-stress group (from each diet). Bracket: significant differences (*p* < 0.05) between dietary groups at the same sampling time (CD, LAN4, and LAN6, respectively). **(A)** TNFα. **(B)** IL-10. **(A,B)** Data obtained from ELISA are expressed as fold change related to the pre-stress group of each diet.

The analysis of cytokines in plasma samples (Figure 2) showed that TNFα secretion (Figure 2A) increased in all groups at 3 h post-stress (CD: 2.15-fold ± 0.38, LAN4: 2.00-fold ± 0.09, LAN6: 1.89-fold ± 0.23), 6 h post-stress (CD: 1.90-fold ± 0.26, LAN4: 1.39-fold ± 0.16, LAN6: 1.76-fold ± 0.12), and 24 h post-stress (CD: 1.79-fold ± 0.12, LAN4: 1.57-fold ± 0.15, LAN6: 1.86-fold ± 0.23) compared to their respective pre-stress group. Comparison between diets showed that LAN4 had significantly lower TNFα levels compared to CD and LAN6 at 6 h post-stress.

In the case of IL-10 (Figure 2B), the results showed that in the CD group, IL-10 levels increased at 6 h post-stress (1.14-fold ± 0.04) and decreases at 0 (0.54-fold ± 0.05), 1 (0.60-fold ± 0.04), 3 (0.82-fold ± 0.09), and 12 h (0.60-fold ± 0.06) post-stress compared to the pre-stress group (1.00-fold ± 0.07).

Whereas LAN4 showed a decrease of IL-10 at 0 (0.51-fold ± 0.04), 1 (0.51-fold ± 0.04), and 12 h (0.55-fold ± 0.07) post-stress (compared to pre-stress group), LAN6 showed increased levels at 3 (1.31-fold ± 0.06), 6 (1.68-fold ± 0.03), and 24 h (1.48-fold ± 0.06) post-stress (compared to the pre-stress group: 1.00-fold ± 0.25). Finally, the analysis by sampling time showed that LAN6 had higher levels of IL-10 than CD and LAN4 (at 0, 1, 3, 12, and 24 h post-stress), and the CD group had higher levels than LAN4 at 6 h post-stress.

### Biomarkers in the Distal Intestine

**FIGURE 3:**
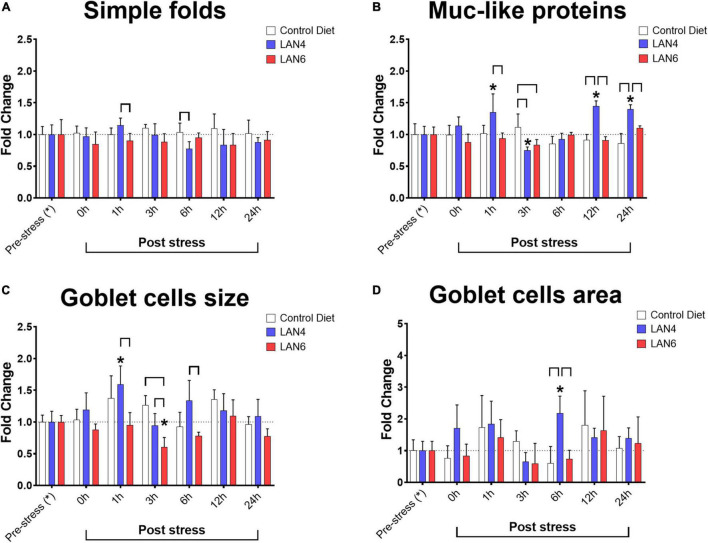
Morphometric of PAS-stained tissue sections and detection of Muc-like proteins in the distal intestine from Atlantic salmon. Sampling times: pre-stress, 0 h (immediately after stress), 1, 3, 6, 12, and 24 h post-stress. In white: CD. In blue: LAN4. In red: LAN6. Each bar: *n* = 4. *: Significant differences (*p* < 0.05) compared with pre-stress group (from each diet). Bracket: significant differences (*p* < 0.05) between dietary groups at the same sampling time (CD, LAN4, and LAN6, respectively). **(A)** Simple folds. **(B)** Muc-like proteins. **(C)** Goblet cell size. **(D)** Goblet cell area. **(A–D)** Data are expressed as fold change related to the pre-stress group of each diet.

Morphometric data (Figure 3) from DI showed significant differences in villi simple fold length between diets at 1 h post-stress (LAN4: 1.14-fold ± 0.11 and LAN6: 0.90-fold ± 0.12) and 6 h post-stress (CD: 1.14-fold ± 0.11 and LAN4: 0.90-fold ± 0.12). Moreover, whereas in the goblet cell area, a significant difference was only detected at 6 h post-stress (increased value in LAN4: 2.18-fold ± 0.53, compared to CD: 0.60-fold ± 0.53 and LAN6: 0.74-fold ± 0.28). Several differences were detected for goblet cell size. In this parameter, an increase in the size of these cells was detected at 1 h post-stress in LAN4 (1.59-fold ± 0.29), compared to the pre-stress group (1.00-fold ± 0.17), and a decrease at 3 h post-stress in LAN6 (0.60-fold ± 0.15), compared to the pre-stress group (1.00-fold ± 0.10). Similarly, the analysis between the different diets showed that in the LAN6 group at 1 (0.95-fold ± 0.20), 3 (0.60-fold ± 0.15), and 6 h (0.78-fold ± 0.06), there was a smaller goblet cell, compared with LAN4 at the same post-stress sampling times (1.59-fold ± 0.29, 0.94-fold ± 0.19, 1.34-fold ± 0.32, respectively), and with CD at 3 h post-stress (1.26-fold ± 0.15). Representative histology images are shown in Supplementary Data Sheet 2.

The detection of Muc-like proteins showed differences in LAN4 at 1 (increase 1.35-fold ± 0.29), 3 (decrease 0.75-fold ± 0.05), 12 (increase: 1.45-fold ± 0.08), and 24 h (increase: 1.40-fold ± 0.07) post-stress compared to baseline levels (pre-stress). Comparison between diets within sampling time showed that CD group had a higher level of Muc-like proteins at 3 h post-stress (1.12-fold ± 0.21) compared to LAN4 (0.75-fold ± 0.05) and LAN6 (0.84-fold ± 0.08). In addition, the LAN4 group had higher levels of these proteins at 1, 12, and 24 h post-stress, compared with CD (at 12 h: 0.91-fold ± 0.09 and 24 h: 0.86-fold ± 0.15), and LAN6 (at 1 h: 0.88-fold ± 0.12, 12 h: 0.91-fold ± 0.06 and 24 h: 1.10-fold ± 0.03).

### Correlations

**FIGURE 4:**
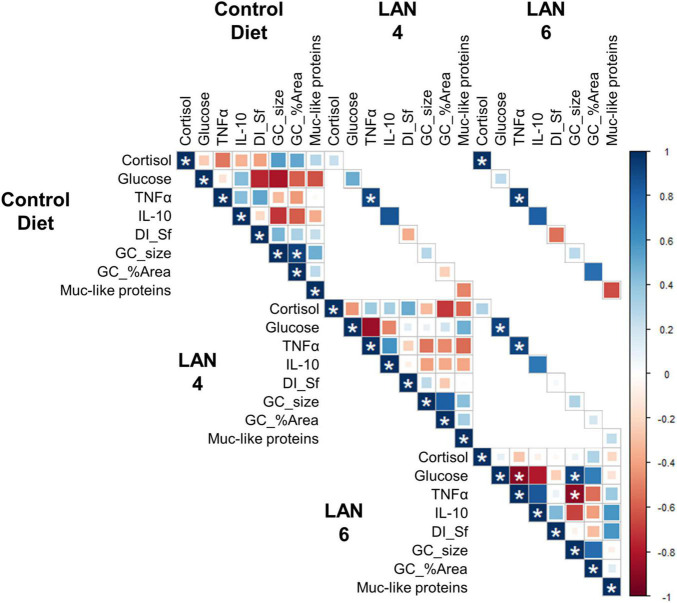
Correlation analysis among the different parameters (cortisol, glucose, TNFα, IL-10, DI simple folds, goblet cells size, goblet cells area, and Muc-like proteins). All the parameters that are significantly correlated (*p* < 0.01) are denoted by *. Degrees of freedom = 6. In blue: positive correlation. In red: negative correlation.

The correlation among the different parameters (Figure 4) showed that cortisol levels in CD and LAN6 were significantly positively correlated (0.98). Moreover, glucose levels in LAN4 and LAN6 were also positively correlated (0.92). Regarding immunological markers, TNFα showed a significant positive correlation among all different diets (CD| L4 = 0.91, CD| L6 = 0.94, L4| L6 = 0.92).

In LAN6, TNFα was significantly negatively correlated with plasma glucose (−0.90) and goblet cell size (−0.90). In addition, goblet cell size was positively correlated with plasma glucose (0.92).

### RNA-Seq in Gills

The comparison between LAN4 and CD showed that among the different sampling time points (pre-stress group, 1 h post-stress, and 6 h post-stress), DEGs were more upregulated than downregulated (Table 4): 238 upregulated DEGs and 125 downregulated DEGs were detected in the pre-stress group. At 1 h post-stress, 73 upregulated DEGs and 33 downregulated DEGs were detected, and at 6 h post-stress, the highest number of modulated DEGs was detected (686 upregulated and 421 downregulated).

**TABLE 4:** Significant differentially expressed genes (DEGs) and term enrichment per comparison.

LAN4 | CD	DEGs		Term Enrichment	
		
	Upregulated	Downregulated	Upregulated	Downregulated
Pre-stress	238	125	130	0
1 h post-stress	73	33	29	0
6 h post-stress	686	421	208	144

The enrichment analysis and functional classification of DEGs in the gills (Table 4) between LAN4 and CD in the pre-stress group showed only upregulated terms (130) in LAN4. The same trend was observed at 1 h post-stress (30 upregulated terms). However, at 6 h post-stress, both upregulated and downregulated terms (208 and 144, respectively) were detected. The complete list of terms (enrichment FDR, number of genes, fold enrichment, and pathways) is shown in Supplementary Data Sheet 1. RNA-seq raw data are available in the Gene Expression Omnibus database (GEO-NCBI: GSE189236).

**FIGURE 5:**
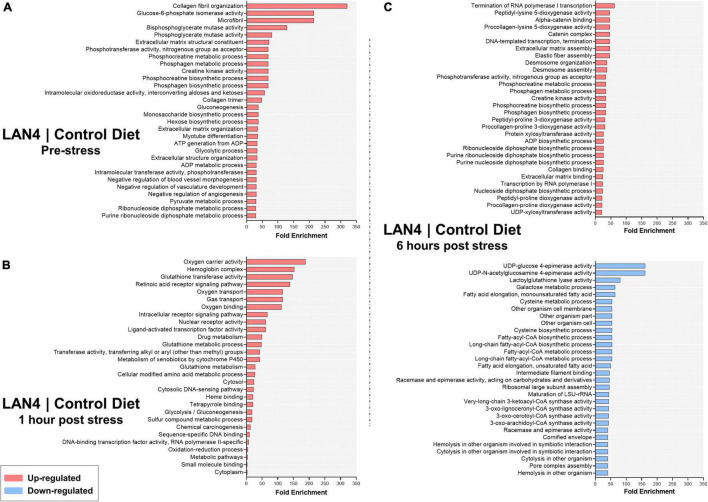
Top #30 terms significantly enriched (minGSSize = 2) in gills from fish fed LAN4 diet compared to fish fed control diet (CD). The list is ordered by fold enrichment. **(A)** Terms in LAN4 compared to CD at the pre-stress group. **(B)** Terms in LAN4 compared to CD at 1 h post-stress. **(C)** Terms in LAN4 compared to CD at 6 h post-stress. In red: upregulated terms. In light blue: downregulated terms.

The top #30 terms per comparison showed that in the pre-stress group (Figure 5A), the upregulated terms (in LAN4) were mostly related to collagen fibril organization, phos/bisphosphoglycerate mutase activity, creatine kinase activity, glycolytic and gluconeogenesis process, metabolic and biosynthetic process, negative regulation of vasculature development, and extracellular matrix structural constituent. At 1 h post-stress (Figure 5B), the LAN4 group showed upregulated terms (compared to CD) associated with oxygen transport and carrier activity, hemoglobin complex, glutathione transferase activity, glutathione metabolic process, intracellular receptor signaling pathway, oxidation-reduction process, ligand-activated transcription factor activity, and sequence-specific DNA binding, among others.

The same analysis for 6 h post-stress samples (Figure 5C) showed that whereas the upregulation of terms was related to collagen, extracellular matrix assembly, termination of RNA polymerase I transcription, creatine kinase activity, metabolic or biosynthetic process, and dioxygenase activity, the downregulated terms were mainly associated with epimerase activity, galactose metabolic process, and fatty acyl-CoA metabolic or biosynthetic process.

## Discussion

Fish farming faces ongoing challenges during production related to multi-stressor conditions such as handling, sub-optimal nutrition, diseases, and environmental conditions. Hence, understanding the use of functional feeds in terms of stress and disease prevention can add to the further development of sustainable aquaculture.

The main goal of this study was to assess the health beneficial effects of functional feeds containing two different hydrolyzed *D. hansenii* yeast-based products and to understand their modulating properties, associated with the regulation and control of stress-related responses during short-term hypoxia. Studies have already shown that microbial ingredients such as yeast cell wall components have the ability to modulate stress responses in fish (Forsatkar et al., 2017; Fuchs et al., 2017; Herrera et al., 2019). However, these effects can vary and are dependent on the yeast origin, processing, and inclusion rate in particular (Reveco-Urzua et al., 2019; Agboola et al., 2021b; Hansen et al., 2021). In this study, we found that adding 0.1% of two different strains (marine and dairy origin) of *D. hansenii* to diets induced distinctive physiological responses after exposure to acute hypoxia stress. Short-term hypoxia increased plasma cortisol levels 1 h post-stress in Atlantic salmon (with a positive correlation between CD and LAN6 dietary groups). This is in accordance with our previous study (Djordjevic et al., 2021a). However, whereas the LAN6 group seems to maintain a cortisol profile similar to CD, in the LAN4 group, we did not observe an increase in cortisol in the post-stress period, although we detected that in the pre-stress group, the cortisol levels in the LAN4 group were higher than in the LAN6 group. These differences raise new questions. For example, acute hypoxia could be a stress condition that stimulates a beneficial or compensatory response for the fish (as occurred in CD and LAN6), that could even benefit the fish against future similar conditions, or the regulation of the host response (as in LAN4 group) can be a way to avoid the energy expenditure that is associated with stress-related responses. In most vertebrates, cortisol is the main circulating glucocorticoid released during a primary stress response. This hormone is critical for mediating the stress response and can induce both behavioral and metabolic pleiotropic effects (Ortega et al., 2021), and it is a classic marker for its evaluation. In Atlantic salmon, the previous studies have shown a typical pattern for this molecule. After acute stress exposure, the basal level of plasma cortisol (less than 100 ng ml^–1^) increases 1–3 h and then decreases to basal levels (Djordjevic et al., 2012, 2021a; Soleng et al., 2019).

In recent years, there is the possibility of mitigating the negative effects of stress and disease susceptibility of fish through the use of feed additives such as functional amino acids (refers to amino acids beyond serving as the building blocks for proteins and peptides, Wu, 2010; Andersen et al., 2016), minerals, and fatty acids (Herrera et al., 2019). Moreover, it has also been observed that β-glucans and other yeast cell wall components can reduce the cortisol levels in zebrafish, turbot, and trout in response to different stressors (Kenari et al., 2013; Forsatkar et al., 2017; Fuchs et al., 2017). Although how these molecules modify and modulate stress response in fish is still unclear, there are indications that functional feed components may have different modes of action inducing multi-systematic responses. Herrera et al. (2019) described that amino acids such as arginine (critical for the production of reactive nitrogen species) can regulate the activation of cellular defense mechanisms in stimulated macrophages in fish.

Furthermore, leucine, isoleucine, and valine (branched-chain amino acids: BCAA) play an important role in regulating protein synthesis in skeletal muscle. Interestingly, it has been reported that fish under stressful situations have shown an increase in proteolytic activity and a decrease in BCAA plasma levels, which suggests that dietary supplementation appears to be a promising strategy to mitigate negative stress effects in fish (Herrera et al., 2019). Tryptophan also plays a role in the regulation of the stress response, since it can be converted to serotonin (5-HT). In the brain, 5-HT is involved in the control of the HPI axis, and in fish, a correlation between 5-HT activity and plasma cortisol levels has been observed. Related to hormone precursors and neurotransmitters (thyroxine, triiodothyronine, epinephrine, norepinephrine, dopamine, and melanin), tyrosine is a common precursor of these hormones and can influence pigmentation development, feed intake, growth performance, immunity, and survival of fish (Herrera et al., 2019). Methionine levels could be modulated by both acute and chronic stressful conditions. This amino acid also plays a role in the antioxidant and immune status of animals as the precursor of cysteine, which is required for the synthesis of glutathione. Glutathione is well known for its reactive species scavenging function, which plays a protective role during stress conditions (Cheng et al., 2015). Minerals (selenium, manganese, and zinc) also can induce stress attenuation, since they are cofactors for essential enzymes related to the control of oxidative stress. The importance of fatty acids during the stress response is associated with the formation of eicosanoids such as prostaglandins, which can modulate the HPI axis activity in fish (Herrera et al., 2019).

The two strains of *D. hansenii* tested differed in origin, chemical composition, and physical characteristics (Table 2). We speculate that some of these differences, such as the amount of β-glucan and/or difference in cell wall thickness or structure, might be the reason for different modulations of stress and immune responses in fish after short-term hypoxia. Stress and the subsequent increase in cortisol have also been found to be negatively correlated with the number of taxa of the gut microbiome (Uren Webster et al., 2020). A possible explanation could be that the addition of prebiotics, which enhances the growth of specific microbiota, prevented the increase in cortisol levels in the blood (Talpur et al., 2014). Although we did not perform this analysis in this study, further studies should investigate the correlation between cortisol and microbiota after different stress exposures in Atlantic salmon fed microbial ingredients. Furthermore, the higher IL-10 production after the hypoxia stress in LAN6 and CD group compared to LAN4 indicates that LAN4 prevents the secretion of IL-10 by an unknown mechanism. IL-10 is a cytokine that acts by modulating processes associated with immunosuppression (Zou and Secombes, 2016).

Usually, glucocorticoids are strong immunosuppressive agents, and immune suppression is observed during high cortisol secretion. In this study, the LAN4 group showed no increase in cortisol and no increase in IL-10 levels. This finding suggests that the LAN4 could be a beneficial functional ingredient during multiple stress events, for example, when fish are fighting against infectious agents or when they are trying to respond efficiently to vaccination (Rodríguez et al., 2016). These situations make it interesting to evaluate *D. hansenii*-based products in future conditions such as chronic stress or after prolonged feeding times. Moreover, this would help to determine whether the regulation of cortisol secretion or the control of immunosuppressive profiles is beneficial or not for the fish in the different life stages of the animal or aquaculture production conditions.

Stress can enhance or suppress the immune response. An increase in TNFα and IL-10 levels in response to nano-encapsulated β-glucans has been reported in Atlantic salmon (Fredriksen et al., 2011). In this study, there was no difference in TNFα levels among diets. However, an increase of TNFα was detected at 3, 6, and 24 h post-stress in all diets (positively correlated), compared to the pre-stress group. This could be a conserved pattern of mobilization of the immune response during stressful events. In gilthead seabream, an *in vitro* study suggested that the adrenocorticotropin hormone (ACTH) can act independently of cortisol, which leads to increased TNFα mRNA levels in head kidney cells (Castillo et al., 2009). Nevertheless, more research is needed to further explain this phenomenon in Atlantic salmon.

In teleosts, mucosa-associated lymphoid tissues (MALTs) such as intestine (GALT) and gill (GIALT) are continually sensing information from the environment to guarantee the homeostasis necessary for fish survival. This important biological process can also be affected by stressor conditions, which impact health and animal welfare (Parra et al., 2015). In Atlantic salmon, it is known that short-term stress and cortisol can modulate the DI immune response (Niklasson et al., 2014; Djordjevic et al., 2021a) and the data from the present study reinforce this hypothesis. Morphometric changes related to goblet cells, which produce mucin proteins that act as a protective lubricant of the mucous layer against possible physical or chemical damage (Hur et al., 2016), were differentially modulated in CD and LAN diet groups. Whereas in the LAN4 group, fish displayed goblet cells with larger size (compared to those of the LAN6 group) and a higher level of DI Muc-like proteins (compared to the other dietary groups), LAN6 showed an opposite pattern, with the smallest goblet cells and a lower level of DI Muc-like proteins. We hypothesize that this response could be related to circulating cortisol since, in higher vertebrates, goblet cells secrete mucins stored in cytoplasmic granules, which results in small and thin goblet cells after stressful conditions (Johansson and Hansson, 2016). Moreover, in rainbow trout, Baumgarner et al. (2013) have described that short-term starvation stress is capable of inducing a decrease in the concentration of inhibitors that protect epithelia from enzymatic damage (e.g., serine proteases), compromising the ability of the mucosa to avoid bacterial infection in the intestine. Therefore, we propose that modulation of goblet cells and Muc-like proteins is a response of the fish to control or prevent intestinal damage after stress.

When analyzing the results, the LAN4 group showed different patterns of plasma biomarkers (no release of cortisol and IL-10) and DI parameters (goblet cells area and Muc-like proteins) compared to CD and LAN6. Stressors conditions can compromise the overall balance in the organism, increasing the demand for resources (e.g., a higher acquisition of food/energy) or inducing several physiological compensations to guarantee homeostasis. These processes can result in maladaptation (although this term is often associated with chronic stress), which indicates that the regulatory mechanisms in the animal have not been able to compensate for the effects of the stressor, which are very relevant in farmed animals, which includes fish subjected to artificial conditions (Balasch and Tort, 2019). To deepen our understanding of the molecular mechanisms behind these differences after hypoxia stress, we performed a transcriptomic analysis of gills using RNA-seq. Fish gills are important for many critical biological functions such as gas exchange, osmoregulation, excretion of nitrogenous waste, pH regulation, and hormone production (Herrero et al., 2018). Gills are also a relevant immune organ, which regulates interactions among the local microbiota and pathogens (Rességuier et al., 2020). In this organ, our data show that at 1 h post-stress, upregulation of genes related to biological pathways such as oxygen binding or transport, hemoglobin complex, oxidation-reduction process, metabolic pathways, and ligand-activated transcription factor activity occurred in fish fed LAN4. This suggests that LAN4 could modulate the response to short-term stress, seeking to guarantee fish homeostasis. In mammals, it has already been reported that during hypoxic conditions, cells activate adaptive responses to match oxygen supply with the metabolic, bio-energetic process and redox demands by hypoxia-inducible factors (Majmundar et al., 2010). The upregulation of glutathione transferase activity and glutathione metabolism in the LAN4 group could also be associated with oxidative stress-tolerance (Peña-Llopis et al., 2003; Srikanth et al., 2013). Furthermore, it is interesting that at 6 h post-stress, the LAN4 group displayed downregulation of fatty acyl-CoA biosynthetic or metabolic process pathways, which could be involved in the modulation of fatty acid oxidation (Kumari, 2018), which prevents oxidative stress by reactive species production (Ly et al., 2017). In fish, Atlantic salmon smolts exposed to an acute stressor (peracetic acid) were able to address oxidative stress (ROS imbalance), mobilizing systemic and mucosal defenses, by increasing the total antioxidant capacity in the plasma and modulating the gene expression of glutathione peroxidase, glutathione reductase, manganese superoxide dismutase, and copper or zinc superoxide dismutase in skin and gills (Soleng et al., 2019).

In summary, our study has shown that nutritional programming by the use of functional feed containing hydrolyzed *Debaryomyces hansenii* yeasts can be an interesting strategy to improve fish welfare in intensive aquaculture. Understanding mucosal responses to functional feeds in target organs such as gills and intestines during short-term stressors can open new avenues for monitoring fish health status. The results in this study showed that the *D. hansenii* yeast-based product (LAN4) was able to regulate the plasma secretion of cortisol and IL-10, in addition to modulating both the production of Muc-like proteins in DI and the gene expression of several metabolic pathways in Atlantic salmon. Based on these findings, we propose that inactivated D. hansenii yeast-based product (LAN4) could be a promising ingredient in functional feeds for salmon to mitigate stress-related responses.

## Data Availability Statement

The datasets presented in this study can be found in online repositories. The names of the repository/repositories and accession number(s) can be found below: https://www.ncbi.nlm.nih.gov/geo/query/acc.cgi?acc=GSE189236.

## Ethics Statement

All animals were treated according to the laws and regulations for experiments on live animals in the EU (Directive 2010/637EU) and Norway (FOR-2015-06-18-761).

## Author Contributions

BM-L and BD conceived the study with extensive inputs from LTM, JO, and MØ. AG, BD, BM-L, CP, and MI performed the experiments and data analysis. LM produced and validated the antibodies. MC produced and characterized the yeasts. LTM and MØ acquired the funds for this investigation. BM-L and BD drafted the manuscript with substantial contributions from all other authors. All authors contributed to the article and approved the submitted version.

## Conflict of Interest

MC was employee in Lallemand SAS. The remaining authors declare that the research was conducted in the absence of any commercial or financial relationships that could be construed as a potential conflict of interest.

## Publisher’s Note

All claims expressed in this article are solely those of the authors and do not necessarily represent those of their affiliated organizations, or those of the publisher, the editors and the reviewers. Any product that may be evaluated in this article, or claim that may be made by its manufacturer, is not guaranteed or endorsed by the publisher.
