# Supplementary material for: Dietary Inclusion of Hydrolyzed Debaryomyces hansenii Yeasts Modulates Physiological Responses in Plasma and Immune Organs of Atlantic Salmon (Salmo salar) Parr Exposed to Acute Hypoxia Stress
Source: Front Physiol. 2022 Mar 28;13:836810. doi: 10.3389/fphys.2022.836810 (PMC8998430; doi:10.3389/fphys.2022.836810)
Supplement: Supplementary file 1 [file Data_Sheet_1.DOCX]

Supplementary Material


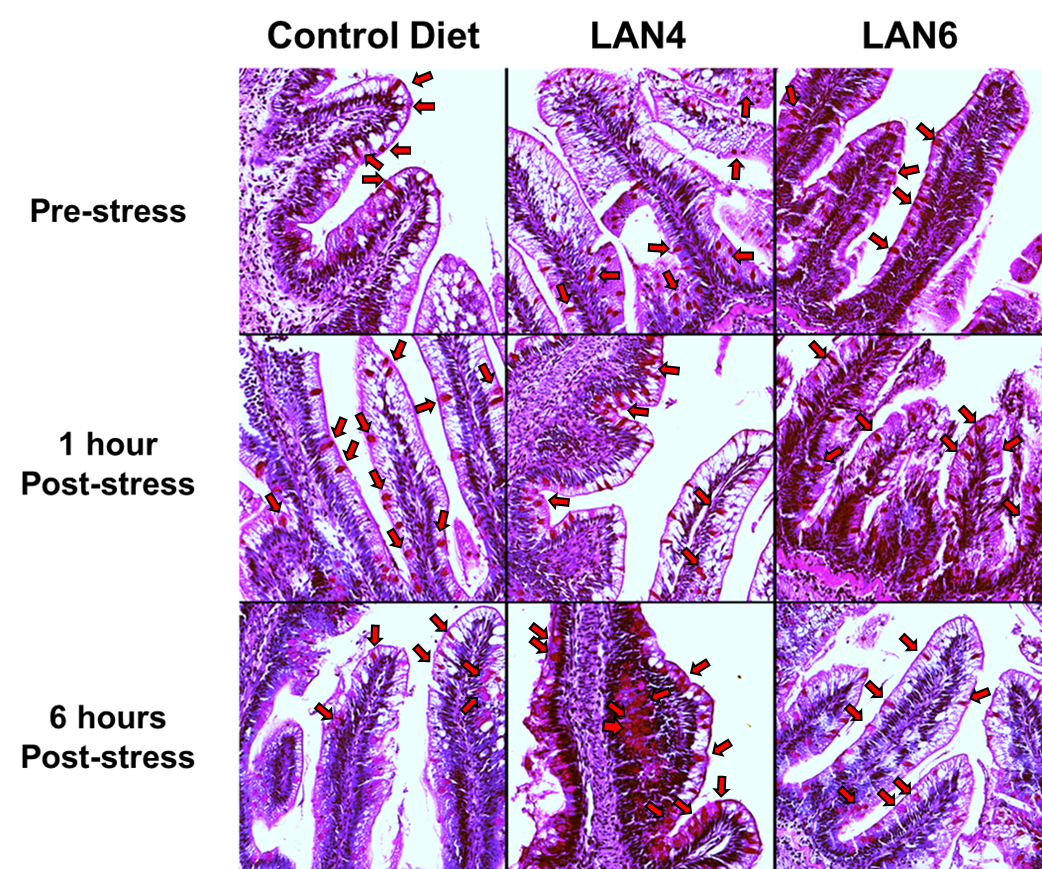


**Supplementary Figure 1.** Periodic acid–Schiff (PAS)-stained tissue sections of DI from Atlantic salmon. Left panels: Control diet. Center panels: LAN4. Right panels: LAN6. In red-brown: PAS+ goblet cells. Arrows: denote goblet cells
